# Different Types of Screen Behavior and Depression in Children and Adolescents

**DOI:** 10.3389/fped.2021.822603

**Published:** 2022-01-24

**Authors:** Tetsuhiro Kidokoro, Akiko Shikano, Ryo Tanaka, Kosuke Tanabe, Natsuko Imai, Shingo Noi

**Affiliations:** ^1^Research Institute for Health and Sport Science, Nippon Sport Science University, Tokyo, Japan; ^2^School of Health and Sport Science, Osaka University of Health and Sport Science, Osaka, Japan; ^3^Faculty of Modern Life, Teikyo Heisei University, Tokyo, Japan; ^4^Faculty of Sport Science, Nippon Sport Science University, Tokyo, Japan

**Keywords:** screen time, 24-h movement guideline, mental health, youth, exercise

## Abstract

The purpose of this study was to examine the associations between different types of screen behavior and depression, taking into account exercise and sleep among children and adolescents. A total of 23,573 Japanese children and adolescents (aged 8–15 years) participated in this cross-sectional study. Different types of screen behavior, weekly exercise time, sleep duration, and prevalence of depression were assessed using a questionnaire. Independent associations between various types of screen behavior and prevalence of depression were examined using logistic regression analyses after adjusting for age, school, sleep duration, exercise time, and other screen behavior types. A two-way analysis of covariance was conducted to examine whether exercise and sleep can attenuate the negative effects of screen behavior. The associations between screen behavior and depression varied by screen behavior types and participant characteristics. More time spent engaging in newer types of screen behavior, including social media, online games, and online videos, was associated with a higher prevalence of depression. In contrast, more time spent on TV was associated with a lower prevalence of depression. Sufficient exercise can lower the prevalence of depression, regardless of the length of time and content of the screen, and its associations were particularly significant for junior high school girls. Sleep was not associated with the prevalence of depression among any participant group except elementary school boys. Our findings suggest that age- and sex-specific intervention strategies that also consider screen-based behavior can effectively lower the risk of depression in children and adolescents.

## Introduction

Depression and its increasing prevalence among children and adolescents (1, 2) is a serious concern. Depression can affect psychological (3), academic (4), and cognitive well-being (5). There is an ongoing debate as to whether the rapid, widespread use of screen devices such as smartphones and portable games is a potential cause for the increased trends of depression (6–9). To date, the evidence regarding screen-based behavior and depression is mixed with negative (10), positive (7, 11), no effect (12), or heterogeneous effects by contents and context of screen time (8, 13) and participants' characteristics (e.g., age and sex) (8, 14, 15).

Screen-based behavior is traditionally assessed in terms of time spent viewing TV, using computers, and playing video games. However, newer types of screen behavior such as watching movies (e.g., YouTube), using social media (e.g., Facebook, Twitter, and Instagram), and online games are emerging among children and adolescents (16). Importantly, evidence suggests that different types of screen behavior are associated differently with depression (8, 10, 17). For example, a recent systematic review of 70 studies showed that computer use and video game playing, but not TV viewing, were significantly associated with depression among youth (8). There are potential mechanisms underlying different effects according to screen behavior types, including displacement hypothesis (18, 19) and upward social comparison hypothesis (20–22). The displacement hypothesis posits that screen time is negatively associated with depression because healthier activities, including exercise and sleep, are displaced by screen behavior types (18, 19). In this scenario, the associations between screen behavior and depression should be equivalent across different screen behavior types because all of them displace time for participating in healthier activities within a 24 h framework (10, 23). In contrast, the upward social comparison hypothesis posits that the effects of screen behavior on depression can vary based on the content viewed on screens (20–22). In particular, upward social comparisons occur when people compare themselves to others who are in more favorable positions (20–22). Previous studies showed that upward social comparison also occurs while using social media because it creates feelings of inferiority (24–26). In this scenario, social media might have larger effects than video games, which do not contain depictions of actual real-life individuals to whom the youth can socially compare themselves (10). In either case, this evidence suggests that viewed contents should also be considered when the association between screen time and depression is examined.

Evidence suggests that sufficient physical activity, sleep duration, as well as low levels of sedentary behavior are all independently associated with better mental health (27–29). Importantly, these behaviors are interrelated and co-dependent (23). Considering these characteristics, Canada released 24 h movement guidelines that recommend children and youth achieve three recommendations (i.e., physical activity, recreational screen time, and sleep) simultaneously (23). A recent systematic review suggests that meeting all three recommendations is preferable to reduce the risk of depression compared to meeting none of the recommendations (30). Additionally, a cross-sectional study with more than 17,000 Canadian children (aged 10–17 years) indicated that those who achieved any given recommendation had better mental health, including life satisfaction, prosocial behavior, and fewer emotional problems than those who did not achieve any of the recommendations (31). While the previous study provided important implications, the authors only evaluated the time spent on TV, videos, DVD, and computer games (31), and they did not specifically evaluate the time spent on newer types of screen behavior including social media and online games that are becoming increasingly popular among children and adolescents (16). To the best of our knowledge, no study has examined the combined association between physical activity (or exercise), sleep, and various types of screen behavior, including traditional and newer screen behavior types, with depression among children and adolescents. Therefore, the purpose of the present study was to examine the associations between various types of screen behavior and depression, taking into account exercise and sleep among children and adolescents.

## Materials and Methods

### Participants

**Figure 1:**
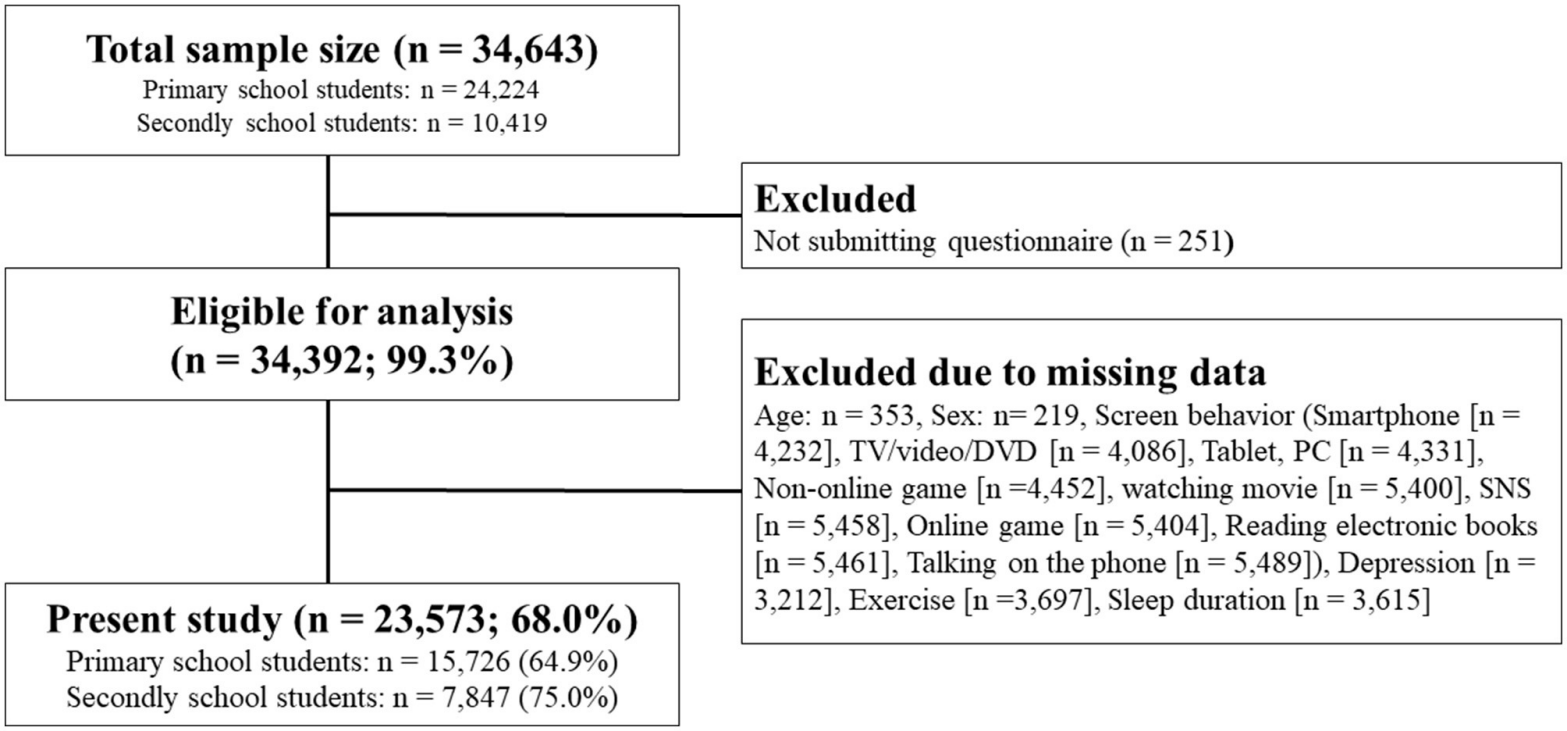
Flow chart of the data sampling.

This cross-sectional study was conducted as a census survey in Setagaya ward, Tokyo, Japan. The details have been described elsewhere (32). In brief, all children and adolescents (aged 8–15 years) from public elementary and junior high schools in the Setagaya ward were invited to participate in the present study. A questionnaire survey was conducted in March 2019 in these schools. The questionnaire for elementary school children was shared with parents who helped with completing it. Junior high school adolescents independently answered the questionnaire. Participants and their parents/guardians were provided with detailed information, including the purpose and contents of the present study. They were also informed about their right to withdraw from the study at any time and were provided with complete assurance regarding the confidentiality of their data. Among the total and eligible samples (*n* = 34,643), 251 were excluded as they did not submit written informed consent (0.7%). Among those who submitted written informed consent (*n* = 34,392; 99.3%), data from 10,819 students (31.2%) were excluded as there was at least one missing piece of demographic data (age and sex), exposure (screen behavior, exercise, and sleep duration) and/or outcome variables (depression). The final sample for the present study comprised 23,573 Japanese children and adolescents (aged 8–15 years) (elementary school students: *n* = 15,726, junior high school students: *n* = 7,847, valid data = 68.0%). Figure 1 presents this sampling process. The present study was approved by the ethics committee of the Nippon Sport Science University (approval No. 015-H075).

### Recreational Screen Behavior

The questionnaire, based on national surveillance (33), asked about recreational (i.e., screen behavior outside of school) screen time. Participants were asked the following four questions about their weekly activity. (1) How long do you watch TV, videos, DVD? (2) How long do you watch online videos? (3) How long do you use social media? (4) How long do you play online games? Participants were asked to report the time spent on each screen behavior in a week.

### Exercise and Sleep Duration

For exercise time, participants were asked the following question: usually, how long do you play sports or exercise outside of school? Here, participants were asked to respond with their exercise time for each day of the week (from Monday to Sunday). Thereafter, the weekly exercise time was calculated by summing the exercise time for each day of the week. The questions were derived from an annual national physical fitness survey among Japanese children and adolescents aged 10–11 and 13–14 years (called “National Survey of Physical Fitness, Athletic Performance and Exercise Habits”) (34, 35). While the questionnaire has not been validated using objective physical activity measures (e.g., accelerometers and doubly labeled water), the time spent on exercise evaluated by the questionnaire has been shown to be significantly associated with health-related outcomes including objective physical fitness performance (34, 35). For sleep duration, participants were asked about their usual weekday wake-up time and bedtime. Thereafter, the sleep duration was calculated by subtracting the wake-up time from the bedtime. Participants were categorized into two groups (meeting sleep guidelines vs. not meeting sleep guidelines) based on the 24 h movement guideline that recommends 9–11 h/day of sleep for 5–13 years old children and 8–10 h/day of sleep for 14–17 adolescents (23).

### Depression

Depression symptoms were measured using a modified version of the depression questionnaire developed by the American Psychiatric Association (36). The modified version of the questionnaire was developed by the Japanese Association of School Health for Japanese school-aged children, and the questionnaire has been tested for its validity and reliability among non-depressed Japanese children and adolescents (33). The questionnaire includes eight questions. Depression is defined using the criteria developed by the Japan Society of School Health (33).

### Statistical Analysis

To examine if any difference existed in age, screen behavior, exercise, sleep, and the prevalence of depression by sex (boys vs. girls) and school type (elementary school vs. junior high school), *t*-tests were performed. Independent associations between various types of screen behavior and prevalence of depression were examined by logistic regression analyses. The presence of depression was considered as a main outcome variable after adjustments were made for age, school, sleep duration, exercise time, and other screen behavior types (e.g., when TV was modeled as the main exposure, the analysis was adjusted for the other three (i.e., online videos, online games, and social media) exposures). To understand the dose-response relationships between screen behavior and depression, we categorized the participants into four groups (“0–30 min,” “30–60 min,” “1–2 h,” and “>2 h”) based on time spent engaging in each screen behavior, with the least usage group (i.e., “0–30 min”) henceforth mentioned as the reference group. Odds ratios (ORs) and 95% confidence intervals (95% CIs) were estimated. To examine whether exercise and sleep can attenuate the negative effects of screen behavior, a two-way analysis of covariance (ANCOVA) was conducted after adjusting for age, school, sleep duration (i.e., when the exercise was modeled as main exposure, the analysis was adjusted for sleep duration), exercise time (i.e., when sleep was modeled as the main exposure, the analysis was adjusted for exercise), and other screen behavior types (e.g., when TV was modeled as the main exposure, the analysis was adjusted for the other three exposures). For exercise, the participants were categorized into two groups (“High EX” vs. “Low EX”) based on the median value of the time spent doing exercise. For sleep, the participants were categorized into two groups (“Met sleep guideline” vs. “Not met sleep guideline”) based on the 24-h movement guideline (23). All statistical analyses were performed using IBM SPSS Statistics for Windows, version 27.0 (IBM Corporation, Armonk, NY, USA).

## Results

### Descriptive Characteristics of the Participants

There were significant differences in screen behavior types by sex (boys vs. girls) and school type (elementary school vs. junior high school) (Table 1). Boys spent more time watching online videos (elementary school students only) and online games than girls. In contrast, the girls spent more time watching TV, videos, DVD, and social media than boys. Junior high school students spent more time on all screen behavior types than elementary school students. Boys had a higher prevalence of depression than girls among elementary school students. Junior high school students had a higher prevalence of depression than elementary school students. Boys spent more time exercising than girls. Boys in junior high school spent less time exercising than boys in elementary school, but the opposite results were found for girls. Among junior high school students, boys spent more time sleeping than girls did. Junior high school students spent less time sleeping than elementary school students (Table 1).

**Table 1:** Descriptive characteristics of the participants.

					**Comparisons**, ***p*****-value**			
	**Elementary school**		**Junior high school**		**Boys vs. Girls**		**Elementary vs. junior**	
	**(***n*** = 15,726)**		**(***n*** = 7,847)**				**high school**	
	**Boys (***n*** = 8,010)**	**Girls** **(***n*** = 7,716)**	**Boys** **(***n*** = 4,189)**	**Girls** **(***n*** = 3,658)**	**Elementary school**	**Junior high school**	**Boys**	**Girls**
**Basic characteristics**								
Age (years)	9.7 ± 1.6	9.3 ± 1.7	14.0 ± 0.8	14.0 ± 0.8	**<0.001**	0.330	**<0.001**	**<0.001**
**Screen behavior**								
TV, video, DVD (min/day)	80.2 ± 57.0	84.5 ± 60.1	98.7 ± 88.7	112.1 ± 94.5	**<0.001**	**<0.001**	**<0.001**	**<0.001**
Watching online video (min/day)	26.6 ± 40.8	25.4 ± 41.9	76.8 ± 80.2	75.7 ± 78.8	**0.019**	0.494	**<0.001**	**<0.001**
Social media (min/day)	1.6 ± 10.6	3.2 ± 13.8	28.5 ± 49.9	54.7 ± 67.3	**<0.001**	**<0.001**	**<0.001**	**<0.001**
Online game (min/day)	18.7 ± 37.7	7.0 ± 20.9	74.1 ± 87.7	22.4 ± 50.6	**<0.001**	**<0.001**	**<0.001**	**<0.001**
**Depression**								
Depressive symptom [% (*n*)]	3.3 (264)	2.7 (208)	9.5 (398)	8.8 (322)	**0.001**	0.245	**<0.001**	**<0.001**
**Exercise and sleep**								
Exercise time (min/day)	74.5 ± 58.9	47.1 ± 45.6	69.3 ± 62.0	52.3 ± 58.6	**<0.001**	**<0.001**	**<0.001**	**<0.001**
Sleep duration (min/day)	545.1 ± 47.0	545.9 ± 49.0	449.4 ± 76.8	436.6 ± 73.5	0.189	**<0.001**	**<0.001**	**<0.001**

*Data are expressed as mean (or percentage) and standard deviation. Bold values represent statistically significant p-values (< 0.05)*.

### Various Types of Screen Behavior and Depression

**Figure 2:**
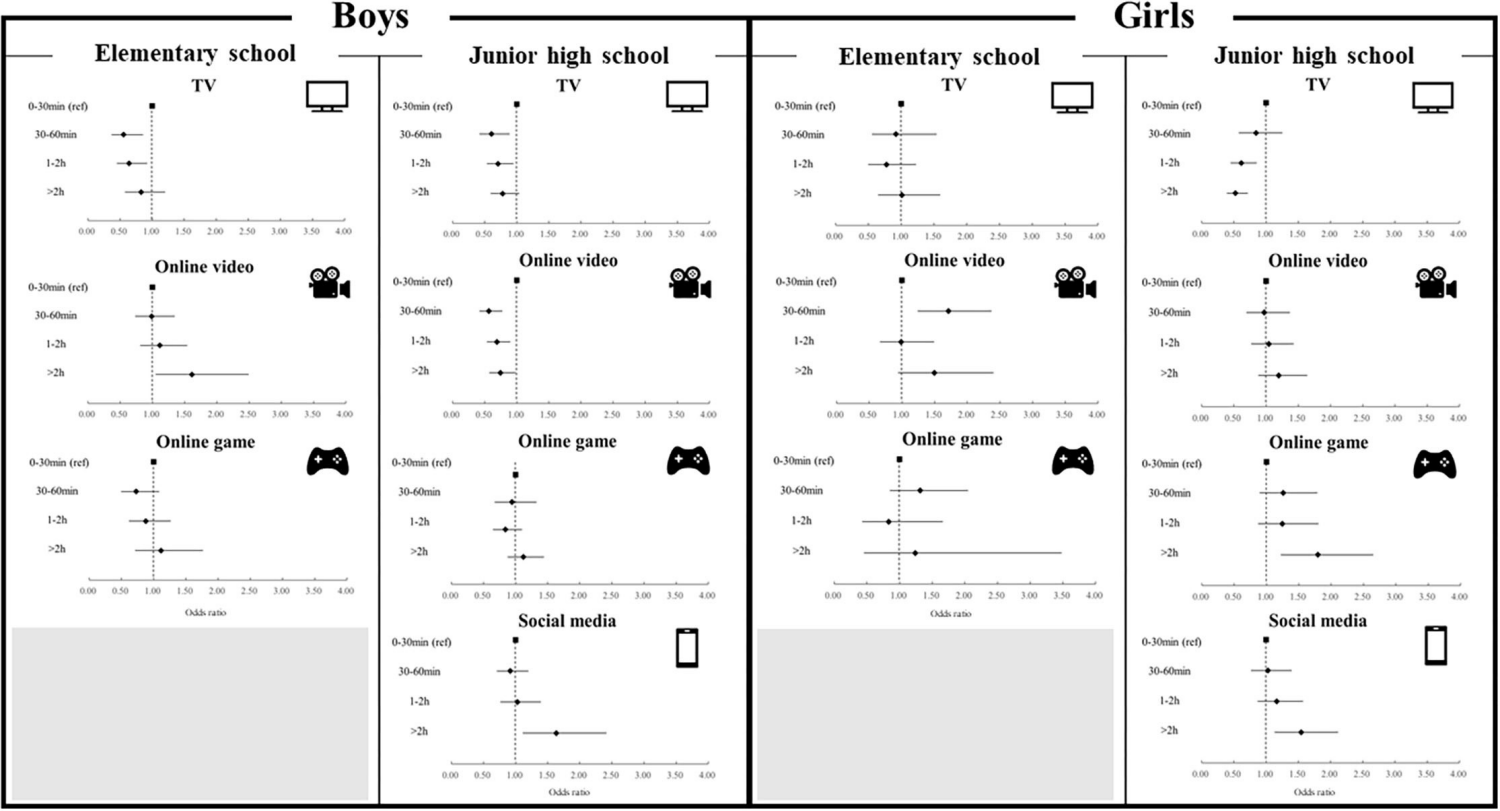
Various types of screen behaviors and depression. Data are expressed as odds ratios and 95% confidence interval. Independent associations between various types of screen behaviors and prevalence of depression were examined by logistic regression analyses with the presence of depression as a main outcome variable after adjusting age, school, sleep duration, exercise time, and other screen behaviors [e.g., when TV was modeled as the main exposure, the analysis was adjusted for the other three exposures (i.e., online videos, online game and social media)].

The associations between screen behavior and depression varied according to screen behavior type (Figure 2). Junior high school students (both boys and girls) who spent more than 2 h/day on social media had a higher prevalence of depression than the reference group. Girls in junior high school who spent more than 2 h/day playing online games had a higher prevalence of depression than the reference group. Additionally, boys in elementary school who spent more than 2 h/day of watching online videos had a higher prevalence of depression than the reference group. However, more time spent watching online videos was associated with a lower prevalence of depression among junior school boys. Furthermore, more time spent watching TV was associated with a lower prevalence of depression among boys and girls.

### Combined Associations of Exercise and Screen Behavior Types With Depression

**Figure 3:**
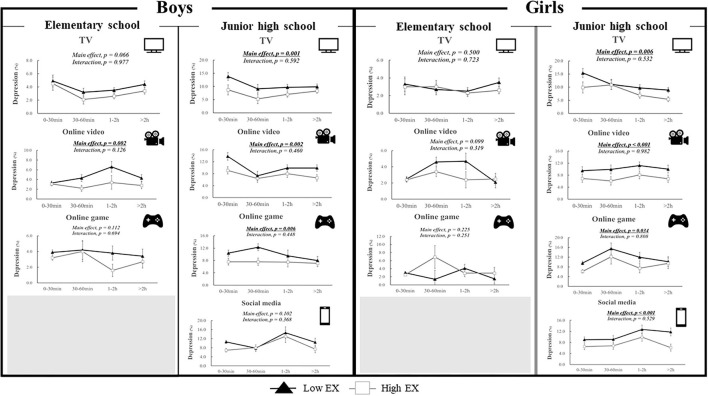
Combined associations of exercise and screen behaviors with depression. Data are expressed as odds ratios and 95% confidence interval. Two-way analysis of covariance (ANCOVA) were conducted after adjusting for age, school, sleep duration and other screen behaviors (e.g., when TV was modeled as the main exposure, the analysis was adjusted for the other three exposures). The participants were categorized into two groups (“High EX” vs. “Low EX”) based on the median value of the time spent in exercise.

Figure 3 shows the results of the two-way ANCOVA analysis (exercise × screen behavior). Exercise significantly affects screen behavior of elementary school boys (online videos), junior high school boys (TV, online videos, and online games), and junior high school girls (all screen behavior types). There were no significant interactions between exercise and screen behavior in relation to the prevalence of depression.

### Combined Associations of Sleep and Screen Behavior Types With Depression

**Figure 4:**
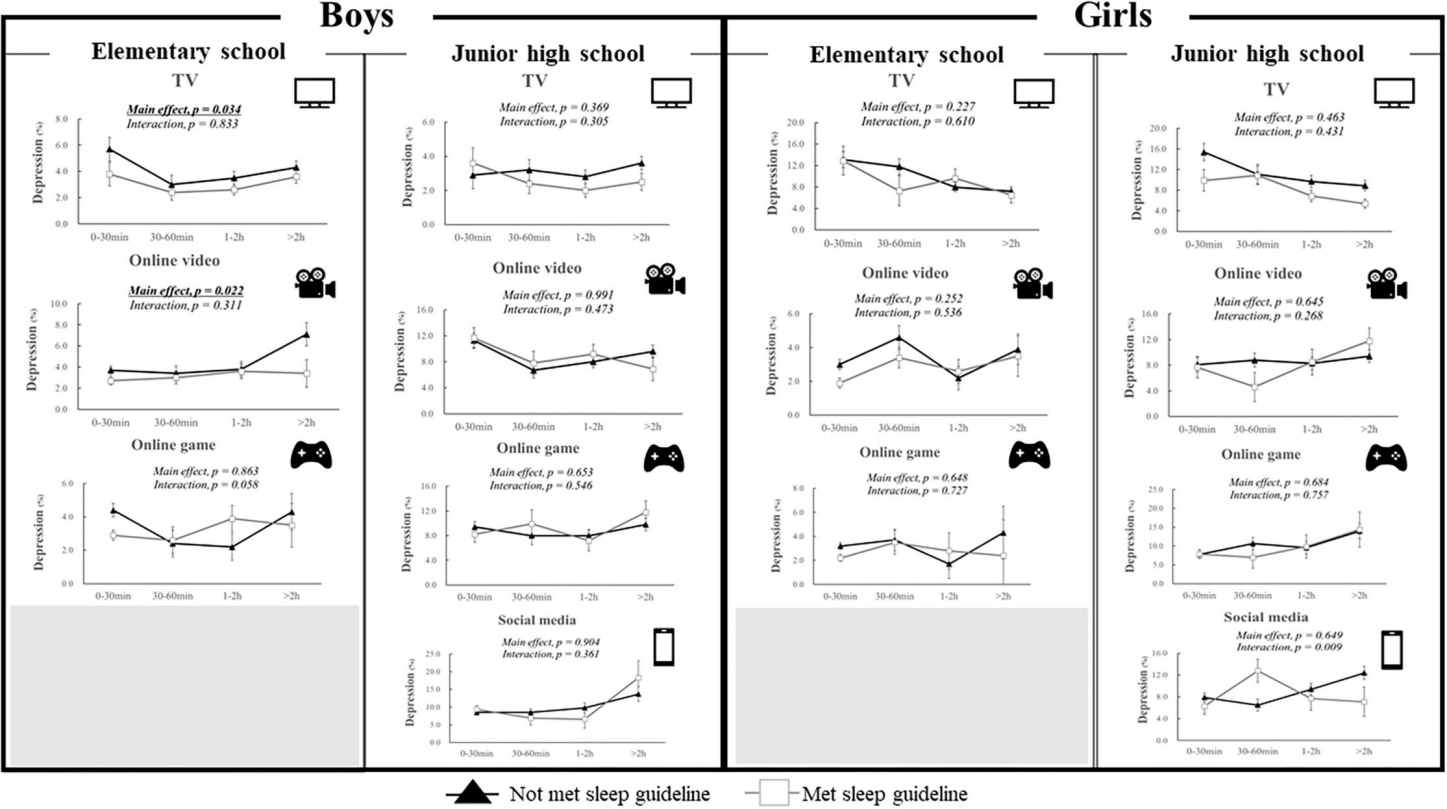
Combined associations of sleep and screen behaviors with depression. Data are expressed as odds ratios and 95% confidence interval. Two-way analysis of covariance (ANCOVA) were conducted after adjusting for age, school, exercise time, and other screen behaviors (e.g., when TV was modeled as the main exposure, the analysis was adjusted for the other three exposures). The participants were categorized into two groups (“Met sleep guideline” vs. “Not met sleep guideline”) based on the 24 h movement guideline (23).

Figure 4 shows the results of the two-way ANCOVA analyses (sleep × screen behavior). There were significant main effects for sleep among elementary school boys (TV and online videos). There were no significant main effects for sleep among girls and junior high school boys.

## Discussion

The present study examined the association between various screen behavior types and depression, while also taking into consideration the influence of exercise and sleep, among a large sample of children and adolescents. The present study revealed that the associations between screen behavior and depression varied by screen behavior types and participant characteristics (sex and developmental stage). In general, more time spent on newer types of screen behavior, including social media, online games (among junior high school girls), and online videos (among elementary school students) was associated with a higher prevalence of depression. In contrast, more time spent on TV, which was traditionally assessed as screen behavior, was associated with a lower prevalence of depression. Sufficient exercise can lower the prevalence of depression, regardless of the length of time and content of screen behavior types, which was particularly significant for junior high school girls. Sleeping for recommended hours can lower the prevalence of depression, but only in elementary school boys. These results could provide important insights to promote psychological health among children and adolescents in the current digital society, where screen devices are ubiquitous (6).

### Explanations of Main Findings

This is the first study to reveal that sufficient exercise (particularly for junior high school students) and sleep (at least for elementary school boys) can attenuate the negative effects of various types of screen behavior on depression in children and adolescents. There are several potential mechanisms that explain how sufficient exercise and sleep are associated with lower prevalence of depression. For example, exercise can reduce the risk of depression through biological (e.g., improving neuroplasticity, neuroendocrine response, inflammation, and oxidative stress) as well as psychological processes (e.g., enhancing self-esteem, social support, and self-efficacy) (37). The present study showed that exercise had greater effects on depression in adolescents, which can be explained by the higher prevalence of depression in adolescents (9.5% and 8.8% for boys and girls, respectively) than in children (3.3 and 2.7% for boys and girls, respectively). This is consistent with findings from previous studies suggesting that exercise is particularly beneficial for adolescents (38). Among junior high school students, exercise significantly attenuated the negative effects of TV, online videos, and online games on depression in both boys and girls. In contrast, exercise significantly attenuated the negative effects of social media on depression in girls but not boys. The sex differences can be explained by the shorter time spent on exercise and longer time spent on social media by girls compared to boys, which are consistent with previous studies (39, 40). Additionally, previous studies reported that there were larger protective effects of exercise on depression in girls than among boys (41, 42). These characteristics might have resulted in the larger protective effects of exercise from prolonged social media on depression among girls. Short sleep duration was negatively associated with brain function (43, 44), brain structure (44), executive function, including working memory and inhibitory control (45), and daytime active behavior due to sleepiness and fatigue (46). Since the Canadian 24 h movement guidelines were released in 2016 (23), numerous pieces of evidence for the combined associations of physical activity, sedentary behavior, and sleep with health outcomes have been published (30). However, most studies on this topic primarily focused on physical health outcomes, and little attention has been paid to mental health outcomes (30, 47). The present study adds to a small body of evidence by demonstrating the potential protective effects of exercise and sleep on depression, along with comprehensive assessments of various types of screen behavior. This is particularly important because types of screen behavior in children and adolescents have become more diverse than ever (16), and assessing only traditional screen time (such as TV viewing and computer gaming) may not be enough for a comprehensive understanding of screen behavior in this population.

### Associations of Screen Behavior and Depression Differ by Screen Behavior Types

There are several potential mechanisms by which types of screen behavior are associated with depression, including the displacement hypothesis (18, 19) and upward social comparison hypothesis (20–22). The present study suggests that the associations between screen behavior and depression differ by screen behavior types. This suggests that there might be, at least in part, mechanisms other than the displacement hypothesis behind the associations. The upward social comparison hypothesis posits that the effects of screen behavior on depression can vary based on the content viewed on screens (20–22). In particular, upward social comparisons occur when people compare themselves to others who are in more favorable positions (20–22). Previous studies have shown that social media use might be particularly detrimental because it creates feelings of inferiority via upward social comparison (24–26), which is consistent with the findings of the present study. This is concerning because the majority of adolescents in today's world use social media and it has become an essential part of their social life (16, 48).

### Strength and Limitations

The present study had several strengths. The large sample size of the present study enabled us to conduct stratified analyses (stratified by developmental stage and sex) taking into account exercise and sleep. Our results suggest that effective approaches to lower the risk of depression should vary according to sex and developmental stage, which have important implications for policy making, clinical practice, and future interventions. Second, we included both traditional types of screen behavior such as TV and newer types such as watching online videos, spending time on social media, and playing online games. This can provide important insights, given that types of screen behavior in today's children and adolescents have become more diverse than ever (16).

Despite the insights provided in our study, some limitations need to be addressed. First, we used a cross-sectional design; therefore, it was not possible to examine the causal relationships between screen behavior and depression. Indeed, it is possible that depressive children and adolescents are less likely to engage in favorable lifestyle behaviors, including exercise and sleep, as previously suggested (49, 50). Second, we used only self-report measures to assess the time spent on screen behavior types, exercise, and sleep, which may lack in precision. Third, the present study did not include any important co-founding variables, including in-person social interactions with friends and family, which should be considered in future studies (51). Fourth, we did not evaluate “how” participants engage in screen behavior. For example, children and adolescents can use social media while being mentally active (e.g., chatting with friends, posting comments, and pictures) or mentally passive (e.g., reading and scrolling through friends' comments and pictures). A recent review argued that there were differential associations between mentally passive and active sedentary behavior and depression (13). Therefore, the detail of “how” the screen is used should be examined in the future.

## Conclusion

The present study revealed that the associations between screen behavior and depression varied according to screen behavior types and participant characteristics. We found that more time spent on newer types of screen behavior, including social media, online games (junior high school girls only), and online videos (elementary school students only) was associated with a higher prevalence of depression. In contrast, more time spent watching TV was associated with a lower prevalence of depression. Our results suggest that a sex- and developmental stage-specific approach should be used to effectively lower the risk of depression among children and adolescents. Additionally, sufficient exercise can lower the prevalence of depression, regardless of the length of time and content of screen behavior types. Furthermore, the protective effects of exercise may be particularly pronounced in junior high school girls. In contrast, sleep was not associated with the prevalence of depression among any participant group except elementary school boys. This study provides important insights into the current digital society, where screen devices have become a part of the social life of children and adolescents.

## Data Availability Statement

The raw data supporting the conclusions of this article will be made available by the authors, without undue reservation.

## Ethics Statement

The present study was approved by the Ethics Committee of the Nippon Sport Science University (approval No. 015-H075). Written informed consent to participate in this study was provided by the participants' legal guardian/next of kin.

## Author Contributions

SN conceptualized the original idea and constructed the methodology. AS, RT, and KT participated in data collection. TK and NI performed statistical analyses. TK wrote the original manuscript. All authors have read and agreed to the published version of the manuscript.

## Funding

This research was supported by the Japan Sport Agency FY2018 grant.

## Conflict of Interest

The authors declare that the research was conducted in the absence of any commercial or financial relationships that could be construed as a potential conflict of interest.

## Publisher's Note

All claims expressed in this article are solely those of the authors and do not necessarily represent those of their affiliated organizations, or those of the publisher, the editors and the reviewers. Any product that may be evaluated in this article, or claim that may be made by its manufacturer, is not guaranteed or endorsed by the publisher.
